# Pregnancy Preferences and Incident Pregnancy in the US

**DOI:** 10.1001/jamanetworkopen.2025.36697

**Published:** 2025-10-09

**Authors:** Brooke W. Bullington, Isabel Muñoz, W. John Boscardin, Corinne H. Rocca

**Affiliations:** 1 Division of Family Planning, Department of Obstetrics and Gynecology, University of Utah, Salt Lake City; 2 Department of Obstetrics, Gynecology and Reproductive Sciences, Advancing New Standards in Reproductive Health, University of California, San Francisco School of Medicine; 3 Division of Epidemiology, University of California, Berkeley School of Public Health; 4 Department of Medicine, University of California, San Francisco School of Medicine; 5 Department of Epidemiology and Biostatistics, University of California, San Francisco School of Medicine

## Abstract

**Importance:**

Examining associations of pregnancy and childbearing desires with incident pregnancy is vital to understanding whether individuals attain their reproductive preferences. Much of the existing population-based research is limited by simplistic measurement of pregnancy desires and retrospective study designs.

**Objective:**

To assess the prospective association of pregnancy preferences with incident pregnancy and to investigate the degree to which people’s attainment of their pregnancy preferences differs by sociodemographic characteristics.

**Design, Setting, and Participants:**

This cohort study used longitudinal data from the Surveys of Women, state-representative prospective studies of reproductive-aged females from 9 US states (Alabama, Arizona, Delaware, Iowa, Maryland, New Jersey, Ohio, South Carolina, and Wisconsin). Participants were recruited between November 2016 and July 2020 and followed up for 2 to 3 years, depending on the state. Eligible participants were aged 18 to 44 years and female. Analyses included participants who, at the start of each 1-year observation period, could experience pregnancy (ie, not pregnant, no tubal ligation, and not infertile). Data were analyzed from May 2024 to April 2025.

**Exposures:**

The primary exposure was preferences about a potential pregnancy within 3 months and childbearing within 1 year, measured using the Desire to Avoid Pregnancy (DAP) scale. Scores were categorized into low, midrange, and high desire to avoid pregnancy.

**Main Outcome and Measures:**

The primary outcome was self-reported incident pregnancy over the year after completing the DAP scale. Differences in incident pregnancy were examined within DAP score grouping by sociodemographic characteristics using model-estimated probabilities of pregnancy.

**Results:**

The sample included 9565 unique participants (3256 participants [weighted percentage, 48%] aged <30 years) who contributed 18 603 annual longitudinal observations. A majority of participants were nulliparous (4066 participants [weighted percentage, 51%]) or lived with a romantic partner (5952 participants [weighted percentage, 59%]). An estimated 3% (95% CI, 2%-4%) of participants with high DAP scores reported pregnancy over a year, compared with 8% (95% CI, 7%-9%) of those with midrange DAP scores and 25% (95% CI, 23%-27%) of those with low DAP scores. The association of DAP score with pregnancy differed by age, parity, prior-year birth, cohabitation with partner, education level, employment, and racial and ethnic identity.

**Conclusions and Relevance:**

In this population-based cohort study of reproductive-aged females, realization of pregnancy preferences varied by sociodemographic characteristics, highlighting reproductive inequities. These results may be used to inform public health efforts to provide contraception, abortion, preconception, and fertility care.

## Key Points

**Question:**

What is the association of pregnancy preferences with incident pregnancy, and are there differences by sociodemographic characteristics?

**Findings:**

In this cohort study of 18 603 longitudinal observations among representative samples of reproductive-aged females in 9 US states, 3% of those who desired to avoid pregnancy became pregnant annually, compared with 25% of those open to pregnancy. Pregnancy, accounting for preferences, differed significantly by age, parity, prior-year birth, cohabitation with a romantic partner, education level, employment, and racial and ethnic identity.

**Meaning:**

These results highlight potential reproductive inequities and may be used to inform public health efforts to provide contraception, abortion, preconception, and fertility care.

## Teaser

This cohort study examines associations of pregnancy preferences (eg, openness to or desire to avoid pregnancy) with incident pregnancy among individuals of reproductive age in the US.

## Introduction

Investigating the association of pregnancy and childbearing desires and incident pregnancy is imperative for understanding the degree to which individuals attain their reproductive preferences and for identifying underlying causes of undesired pregnancy. Further, assessing how attainment of pregnancy preferences differs by sociodemographic characteristics can identify inequities in reproductive autonomy.

Research examining these associations, however, has been hindered by study design and measurement challenges. Population-based estimates of US pregnancy rates by degree to which individuals desired pregnancy rely on retrospective data and piece together data across sources.^1^ Without information on the pregnancy preferences of individuals who do not experience pregnancy, these data cannot be used to estimate proportions experiencing pregnancy within pregnancy desire grouping (eg, pregnancies among those who do not desire pregnancy).^2^ Additionally, the construct of pregnancy desires or preferences is complex and hard to capture quantitatively.^3,4,5^ Pregnancy desires are often categorized simplistically (eg, wanted vs unwanted) and rarely incorporate a range of preferences, including uncertainty and ambivalence. Until recently, no instrument had been formally developed to measure pregnancy preferences in a way that accurately captures nuances in how people feel about becoming pregnant. Population-based, prospective surveys are rare and costly, limiting inclusion of multi-item measures.

The Desire to Avoid Pregnancy (DAP) scale is a robust psychometric measure of prospective pregnancy and childbearing preferences that addresses many of these limitations.^6^ The scale’s items refer to multiple cognitive, affective, and life consequence considerations and specify a short time frame (3 months for pregnancy and 1 year for childbearing) acknowledging that preferences about pregnancy evolve with changing life circumstances. The instrument accounts for the fact that many people do not hold clear intentions for future pregnancy, allowing for ambiguity and/or uncertainty. With the DAP scale, researchers can more accurately capture pregnancy preferences and examine their associations with behavioral and reproductive outcomes, including incident pregnancy. We purposefully refer to pregnancy preferences instead of intentions to more accurately describe the latent feelings some individuals hold about a potential pregnancy.

We leveraged novel population-based, longitudinal data collected among reproductive-aged females across 9 diverse US states to assess the prospective association of pregnancy preferences with experience of incident pregnancy. While several studies have examined how fertility preferences, including as measured with the DAP scale, are associated with near-term pregnancy outcomes,^7,8,9,10,11^ we are unaware of other population-based studies that have used the instrument. Then, to shed light on potential inequities in reproductive autonomy and inform efforts to improve contraceptive, abortion, fertility, and preconception care, we further examined whether the association of pregnancy preferences with pregnancy differs across sociodemographic groups. Our research questions were: What is the association of pregnancy preferences with incident pregnancy, and how does obtainment of preferences differ across sociodemographic groups? We hypothesized that preferences would be associated with pregnancy, and that these associations would differ significantly across sociodemographic groups.

## Methods

This study was classified as Category 4 exempt by the University of California, San Francisco institutional review board because the data were provided to the research team as a deidentified dataset; thus, no informed consent was needed. This research adheres to the Strengthening the Reporting of Observational Studies in Epidemiology (STROBE) reporting guideline for cohort studies.

### Data Source

We analyzed longitudinal data from the Surveys of Women, state-representative studies of reproductive-aged females in Alabama, Arizona, Delaware, Iowa, Maryland, New Jersey, Ohio, South Carolina, and Wisconsin between November 2016 and June 2023. These surveys were initially developed to evaluate contraceptive access initiatives in some states and then expanded to investigate reproductive health policy changes in others. Details of the survey methodology have been published previously and were similar across states.^12^ Survey administrations were staggered over time; for instance, wave 1 in Delaware and Maryland was in 2016, while wave 1 was in 2020 in Arizona, New Jersey, and Wisconsin (eTable 1 in Supplement 1).

Surveys were fielded and data were managed by NORC at the University of Chicago. The sampling frame for each state’s baseline survey was the US Postal Service computerized delivery sequence file. Census tracks with higher proportions of racial and ethnic minority households and lower population density were oversampled. NORC used a multimode data collection approach, including mailings and follow-up for nonresponse. Data were collected via a self-administered web-based or paper questionnaire.

Participants were eligible for inclusion if they: (1) were aged 18 to 44 years and (2) self-identified as female in Alabama, Delaware, Iowa, Maryland, Ohio, and South Carolina or identified as female, transgender, gender expansive, or something else in Arizona, New Jersey, and Wisconsin. The study consisted of four waves of data collection for each state: a baseline (wave 1), 2 or 3 follow-up surveys (waves 2-4), and a cross-sectional endline survey (wave 5). Follow-up surveys were administered approximately every 1 to 2 years.

### Measures

Our independent variable was pregnancy and childbearing preferences (pregnancy preferences moving forward), measured using the DAP scale.^6^ The scale is comprised of 14 items that assess respondents’ feelings about a potential pregnancy in the next 3 months (eg, “thinking of becoming pregnant makes me feel excited”) and, correspondingly, having and raising a child within the next year (eg, “having a baby would be bad for my life”). Participants respond to each item on a 5-point Likert scale (strongly agree to strongly disagree). Scores range from 0 to 4, with 0 indicating the greatest openness to the prospect of pregnancy and 4 indicating the strongest desire to avoid pregnancy. For incident pregnancy analyses, we used a categorical variable (low DAP, ≤1.5; midrange DAP, >1.5 to ≤2.5; high DAP, >2.5). Our primary outcome was a binary indicator of incident pregnancy, or whether a participant had become pregnant at least once in the last 12 months, measured via self-report on annual follow-up surveys.

We included sociodemographic characteristics we hypothesized might modify the association of DAP score with incident pregnancy.^13,14,15,16,17,18,19^ These included time-varying age group, parity, cohabitation with romantic partner, highest education level, health insurance status, prior-year birth, current employment, self-rated health, baseline religiosity, and self-identified racial and ethnic identity. Race and ethnicity categories included Asian, non-Hispanic; Black, non-Hispanic; Hispanic; White, non-Hispanic; and multiracial or another race or ethnicity. For analyses, racial and ethnic groups with fewer than 1% were included as multiracial or another race, which included Native American, Alaska Native, or American Indian; Native Hawaiian or Pacific Islander; and any race or ethnicity not otherwise specified.

### Statistical Analysis

Analyses included data collected at waves 1 to 4. The DAP scale was not available at the time of baseline survey administration in Delaware and Maryland (2016), so we excluded wave 1 data from those states. Our analysis sample included participants who could experience pregnancy (not pregnant, had not had a tubal ligation, or not infertile) at the wave at which pregnancy preferences were measured.

To describe how pregnancy preferences differed by sociodemographic characteristics, we fit a series of mixed-effects linear regression models with characteristics as the exposure and continuous DAP score as the outcome. We present estimated mean DAP scores and 95% CIs based on these models.

To model the association of pregnancy preferences with incident pregnancy, we used unadjusted mixed-effects logistic regression. DAP score was measured at the survey wave prior to incident pregnancy (ie, wave 1 DAP score and wave 2 pregnancy). We used DAP score groupings to facilitate interpretation of probabilities of pregnancy generated from the models. We considered pregnancy among participants with high DAP scores, and not experiencing pregnancy among participants with low DAP scores, as indicative of not attaining one’s pregnancy preference.

To identify sociodemographic differences in incident pregnancy within levels of pregnancy preferences, we fit a series of mixed-effects logistic regression models. For each sociodemographic characteristic, we fit a separate model with interaction terms between that characteristic and DAP grouping. Time-varying characteristics were measured at the same wave as DAP scores (the survey prior to incident pregnancy). We derived probabilities of pregnancy and 95% CIs from these models and assessed differences in incident pregnancy within each DAP grouping. We present probabilities of pregnancy as percentages throughout.

We applied time-varying survey weights in all models. Sampling weights were assigned by NORC using a 5-step weighting process, which included base sampling weights; adjustment for unknown eligibility, nonresponse, and household size; and poststratification.^12^ Additional weights at waves 2 to 4 were applied to account for loss to follow-up. When modeling DAP score as an outcome, we applied weights from the survey at which DAP was measured. For incident pregnancy models, we applied weights from the survey at which incident pregnancy was measured.

All models accounted for clustering by participant, used robust standard errors to calculate confidence intervals, and included state fixed effects and an indicator of whether data were collected before or after the onset of the COVID-19 pandemic (March 15, 2020).^20^ We initially included state as a random effect but were unable to obtain model convergence due to computational complexity and minimal variation by state (intraclass correlation for state in unweighted models with state and participant as random effects, 0.003). We conducted robustness checks to assess the impact that treating state as fixed effects might have on results. We compared results from (1) unweighted models with both state and participant as random effects with unweighted models with state fixed effects and a participant random effect and (2) weighted models with state and participant as robust clusters with weighted models with state fixed effects and participant as a robust cluster. Results were nearly identical across approaches, providing confidence that treating state as fixed effects would yield similar results as it would have as a random effect.

To allow for comparability with national estimates based on retrospective pregnancy orientation assessment among National Survey of Family Growth (NSFG) respondents, birth vital statistics, and health facility–reported abortion rates,^1,21^ we report the weighted proportion of all pregnancies that occurred among those with low, midrange, and high DAP scores by wave. We also report the weighted proportion of participants who experienced a low, midrange, or high DAP pregnancy by dividing the number of pregnancies among participants in each DAP grouping by the total number of participants by wave.

All analyses were conducted in Stata 17.0 (StataCorp) from May 2024 to April 2025. We assessed statistical significance at α = .05.

## Results

**Figure 1.**
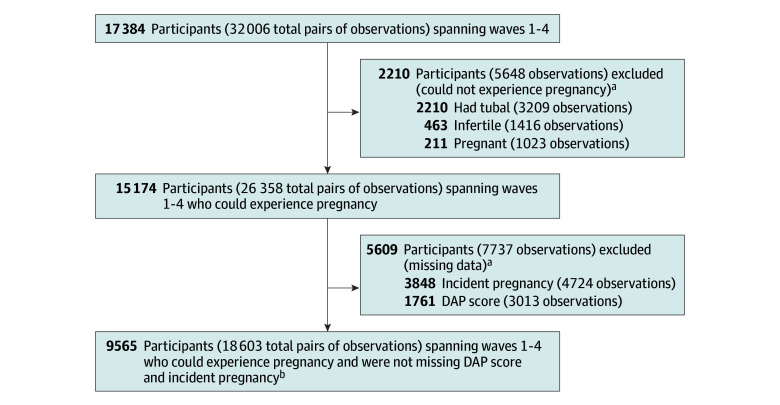
Flowchart of the Analytic Sample With Exclusion Criteria DAP indicates Desire to Avoid Pregnancy scale. ^a^Exclusions were applied sequentially. Each exclusion accounts for prior exclusions. ^b^The final analysis sample included 7396 paired observations from waves 1 to 2, 7488 from waves 2 to 3, and 3719 from waves 3 to 4.

Our analytic sample included 9565 unique reproductive-aged participants (weighted = 5 443 192 individuals), who contributed 18 603 paired longitudinal observations (mean [SD], 1.94 [0.79] paired observations per participant) (Figure 1). Almost one-half of the sample (3256 participants [weighted percentage, 48%]) was younger than 30 years at baseline, and 4066 (weighted percentage, 51%) were nulliparous (Table 1). Most participants (5952 participants [weighted percentage, 59%]) reported living with a partner. Among participants, 8349 (weighted percentage, 84%) had at least some college education, 8571 (weighted percentage, 92%) had health insurance, and 7341 (weighted percentage, 74%) were employed. Approximately one-third of participants (3035 participants [weighted percentage, 34%]) reported religion was not important or that they were not religious. Among participants, 259 (weighted percentage, 4%) were Asian, non-Hispanic; 925 (weighted percentage, 14%) were Black, non-Hispanic; 838 (weighted percentage, 12%) were Hispanic; 7162 (weighted percentage, 66%) were White, non-Hispanic; and 369 (weighted percentage, 5%) were multiracial or another race or ethnicity. Most participants (8959 [weighted percentage, 94%]) reported they were in excellent, very good, or good health.

**Table 1.** Baseline Sociodemographic Characteristics of Reproductive-Aged Females From 9 US States in the Surveys of Women^a^

Characteristic	Unweighted No./total No. (weighted %) (unweighted N = 9565; weighted N = 5 443 192)
Age group, y^b^	
18-24	1525/9553 (26)
25-29	1731/9553 (22)
30-34	2023/9553 (21)
35-39	2133/9553 (16)
40-44	2141/9553 (14)
Parity	
Nulliparous (0)	4066/9455 (51)
Primiparous (1)	1601/9455 (17)
Multiparous (2)	2198/9455 (18)
Multiparous (3)	1014/9455 (9)
Multiparous (≥4)	576/9455 (5)
Prior-year birth	
Yes	636/9445 (7)
No	8809/9445 (93)
Lives with partner	
Yes	5952/9035 (59)
No	3083/9035 (41)
Highest level of education^b^	
High school, equivalent, or less	1204/9553 (17)
Some college	3126/9553 (42)
Bachelor’s degree or more	5223/9553 (41)
Employment^b^	
Employed	7341/9553 (74)
Unemployed	597/9553 (8)
Out of work force	1615/9553 (18)
Race and ethnicity^b^	
Asian, non-Hispanic	259/9553 (4)
Black, non-Hispanic	925/9553 (14)
Hispanic	838/9553 (12)
White, non-Hispanic	7162/9553 (66)
Multiracial or another race or ethnicity^c^	369/9553 (5)
Importance of religion in life (religiosity)	
Very important	3425/9397 (34)
Somewhat important	2937/9397 (32)
Not important	3035/9397 (34)
Self-rated health	
Excellent, very good, or good	8959/9555 (94)
Fair or poor	596/9555 (6)
Has health insurance	
Yes	8571/9340 (92)
No	769/9340 (8)
State	
Alabama	1136/9565 (10)
Arizona	1117/9565 (14)
Delaware	464/9565 (1)
Iowa	1475/9565 (7)
Maryland	469/9565 (7)
New Jersey	1129/9565 (16)
Ohio	1517/9565 (25)
South Carolina	1133/9565 (10)
Wisconsin	1125/9565 (11)

^a^ The table presents baseline (wave 1) characteristics of all participants in the analysis sample, regardless of the wave at which they entered the analysis sample.

^b^ Missing values for age, education level, employment, and race and ethnicity were imputed using hot-deck imputation.^12^

^c^ Racial and ethnic groups with fewer than 1% were included in multiracial or another race, which included Native American, Alaska Native, or American Indian; Native Hawaiian or Pacific Islander; and any race or ethnicity not otherwise specified.

**Figure 2.**
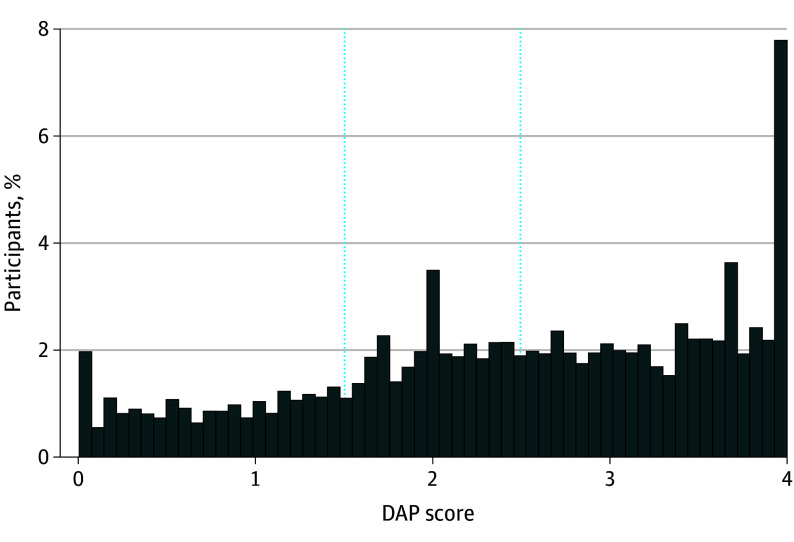
Weighted Desire to Avoid Pregnancy (DAP) Scale Scores at Baseline Histogram showing distribution of weighted DAP scores at baseline for 9565 individuals. Scores range from 0 to 4, with higher scores indicating stronger preferences to avoid pregnancy. Vertical lines represent cut points between low, midlevel, and high DAP score categories.

At baseline, median (IQR) DAP score was 2.50 (1.71-3.43) and mean DAP score was 2.44 (95% CI, 2.40-2.47) (Figure 2). Across all waves, 3856 observations (weighted percentage, 21%) were among participants who reported a low DAP score, 5714 observations (weighted percentage, 31%) were among participants who reported a midrange DAP score, and 9033 observations (weighted percentage, 49%) were among participants who reported a high DAP score.

**Figure 3.**
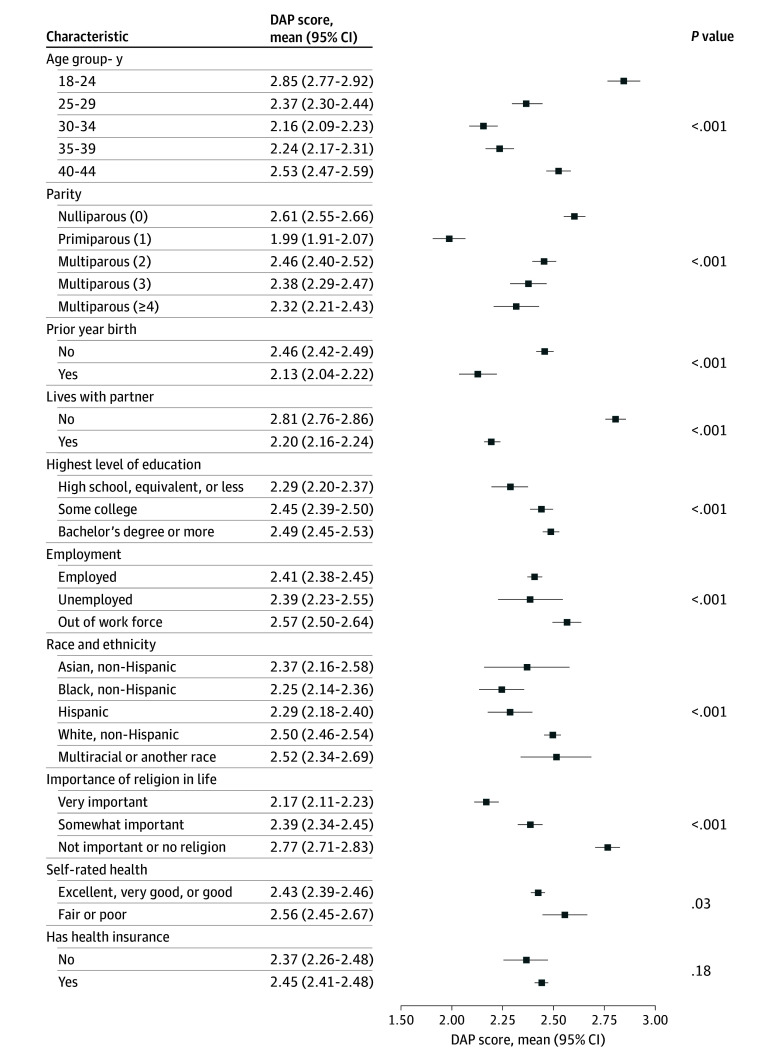
Estimated Mean Desire to Avoid Pregnancy (DAP) Score by Sociodemographic Characteristics Estimated mean DAP scores and 95% CIs by sociodemographic characteristics generated from mixed-effects linear regression models accounting for state and whether data were collected before or after the COVID-19 pandemic. Estimates account for survey weights and clustering by participant; robust standard errors were calculated for the 95% CIs. Scores range from 0 to 4, with higher scores indicating stronger preferences to avoid pregnancy. Racial and ethnic groups with fewer than 1% were included in multiracial or another race, which included Native American, Alaska Native, or American Indian; Native Hawaiian or Pacific Islander; and any race or ethnicity not otherwise specified.

Pregnancy preferences differed significantly by most sociodemographic characteristics (Figure 3). The highest DAP scores were observed among participants aged 18 to 24 years (mean score, 2.85; 95% CI, 2.77-2.92), those not living with a partner (mean score, 2.81; 95% CI, 2.76-2.86), and those of no or low religiosity (mean score, 2.77; 95% CI, 2.71-2.83). The lowest DAP scores were observed among primiparous participants (mean score, 1.99; 95% CI, 1.91-2.07).

There were 1313 participants (unweighted percentage, 14%) who reported experiencing an incident pregnancy during the study period (between waves 1-2, 2-3, 3-4, or across multiple waves). The model-based annual probability of pregnancy was 9% (95% CI, 8%-10%). We found significant differences in probability of pregnancy by DAP score grouping. The probability of pregnancy was 25% (95% CI, 23%-27%) among those with a low DAP score, 8% (95% CI, 7%-9%) among those with a midrange DAP score, and 3% (95% CI, 2%-4%) among those with a high DAP score.

Probability of pregnancy by DAP grouping differed significantly by age group, parity, prior-year birth, education, employment, cohabitation status, and racial and ethnic identity (Table 2 and the eAppendix in Supplement 1). We found no differences according to religiosity, health insurance status, or self-rated health. Associations were largely consistent across states (eTable 2 in Supplement 1).

**Table 2.** Unadjusted Model Percentages of Incident Pregnancy Over 1 Year by DAP Scale Score and Selected Sociodemographic Characteristics^a^

Characteristic	Incident pregnancy by DAP score range, estimated probability (95% CI), %					
	Low score (0-1.5)	*P* value	Midrange score (>1.5-2.5)	*P* value	High score (>2.5-4)	*P* value
Total sample	25 (23-27)	NA	8 (7-9)	NA	3 (2-4)	NA
Age group, y^b^						
18-24	32 (24-39)	Reference	13 (8-17)	Reference	3 (2-4)	Reference
25-29	32 (26-37)	.95	14 (10-18)	.74	5 (3-7)	.04
30-34	28 (24-32)	.35	10 (7-12)	.25	5 (3-7)	.02
35-39	19 (15-24)	.004	4 (3-6)	.001	2 (1-3)	.15
40-47	8 (3-13)	.001	1 (0-2)	.001	1 (0-1)	.003
Parity						
Nulliparous (0)	21 (18-24)	Reference	6 (4-7)	Reference	2 (1-3)	Reference
Primiparous (1)	32 (28-37)	.001	11 (8-14)	.001	8 (5-11)	.001
Multiparous (2)	28 (22-33)	.04	8 (5-11)	.15	3 (2-4)	.13
Multiparous (3)	14 (8-20)	.07	7 (3-11)	.51	4 (2-6)	.04
Multiparous (≥4)	23 (12-34)	.69	12 (5-19)	.03	7 (2-13)	.001
Prior-year birth						
No	23 (21-25)	Reference	6 (5-7)	Reference	3 (2-3)	Reference
Yes	48 (38-57)	.001	26 (19-33)	.001	13 (8-17)	.001
Lives with partner						
No	14 (10-18)	Reference	6 (4-8)	Reference	3 (2-3)	Reference
Yes	28 (25-31)	.001	9 (7-11)	.03	3 (2-4)	.29
Highest education						
≤High school degree or equivalent	24 (18-30)	Reference	11 (7-15)	Reference	5 (3-7)	Reference
Some college	22 (18-25)	.49	8 (6-10)	.07	4 (3-5)	.35
Bachelor’s degree or more	29 (26-32)	.16	7 (5-8)	.01	2 (1-3)	.002
Employment						
Employed	25 (22-27)	Reference	8 (6-9)	Reference	3 (2-4)	Reference
Unemployed	25 (15-36)	.92	9 (4-15)	.49	2 (2-9)	.06
Out of work force	24 (18-30)	.76	10 (6-13)	.23	2 (1-3)	.02
Race and ethnicity						
Asian, non-Hispanic	27 (12-42)	.87	2 (0-4)	.02	5 (0-10)	.19
Black, non-Hispanic	18 (12-24)	.01	12 (7-16)	.02	6 (3-8)	.001
Hispanic	19 (13-25)	.01	8 (4-12)	.72	4 (2-7)	.04
White, non-Hispanic	28 (26-31)	Reference	7 (6-8)	Reference	2 (2-3)	Reference
Multiracial or another race or ethnicity^c^	24 (12-36)	.53	12 (4-20)	.11	1 (0-3)	.24
Importance of religion (religiosity)						
Very important	23 (20-27)	Reference	7 (6-9)	Reference	4 (3-5)	Reference
Somewhat important	26 (22-30)	.25	9 (6-12)	.97	3 (2-4)	.10
Not important	27 (22-32)	.73	8 (5-10)	.39	3 (2-3)	.25
Self-rated health						
Excellent, very good, or good	25 (23-28)	Reference	8 (7-9)	Reference	3 (3-4)	Reference
Fair or poor	19 (11-26)	.12	9 (3-15)	.67	2 (1-4)	.37
Has health insurance						
No	20 (12-29)	Reference	7 (4-11)	Reference	3 (1-5)	Reference
Yes	25 (23-28)	.29	8 (7-9)	.71	3 (2-4)	.85

Abbreviations: DAP, Desire to Avoid Pregnancy Scale; NA, not applicable.

^a^ In 167 participants (0.9% of observations), multiple pregnancies over the course of 1 year were reported. Pregnancies ending in abortion may have been underreported. See the eAppendix in Supplement 1 for further interpretation.

^b^ At baseline, all participants were aged 44 years or younger. At follow-up surveys, some participants were aged 45 to 47 years.

^c^ Racial and ethnic groups with fewer than 1% were included as multiracial or another race, which included Native American, Alaska Native, or American Indian; Native Hawaiian or Pacific Islander; and any race or ethnicity not otherwise specified.

Specifically, for some traits, probability of pregnancy differed consistently across DAP groupings. For example, the probabilities of pregnancy among participants with low, midrange and high DAP scores were highest (and generally similar) among those aged 18 to 24 years, 25 to 29 years, and 30 to 34 years; probability of incident pregnancy declined with increasing age across all DAP groupings. Similarly, the probability of pregnancy across DAP groups was higher among primiparous participants compared with nulliparous and multiparous participants. Participants who had a prior-year birth had higher probability of incident pregnancy across all DAP groupings compared with participants who did not have a prior-year birth.

For other traits, only those with high DAP scores had differences in probability of pregnancy. Among those with a high DAP score, participants with a bachelor’s degree or more had lower probability of pregnancy compared with those with a high school degree, equivalent, or less (2%; 95% CI, 1%-3% vs 5%; 95% CI, 3%-7%). Further, participants with high DAP scores who were out of the work force had a lower probability of pregnancy (2%; 95% CI, 1%-3%) compared with employed participants (3%; 95% CI 2%-4%).

The association of DAP with pregnancy differed only among those with low or midrange DAP scores by cohabitation. Participants who lived with their partner had a higher probability of pregnancy compared with participants who did not live with their partner (low: 28%; 95% CI, 25%-31% vs 14%; 95% CI, 10%-18%; midrange: 9%; 95% CI, 7%-11% vs 6%; 95% CI, 4%-8%).

For racial and ethnic identity, among those with high DAP scores, Black, non-Hispanic participants (6%; 95% CI, 3%-8%) and Hispanic participants (4%; 95% CI, 2%-7%) had higher probability of pregnancy compared with White, non-Hispanic participants (2%; 95% CI, 2%-3%). Among those with low DAP scores, Black, non-Hispanic participants (18%; 95% CI, 12%-24%) and Hispanic participants (19%; 95% CI, 13%-25%) were less likely to experience pregnancy compared with White, non-Hispanic participants (28%; 95% CI, 26%-31%).

Over each annual wave, of pregnancies that occurred (total unweighted, 1488 pregnancies), more than one-half (weighted percentages, 55%-60%; wave 1-2: 382 of 633 pregnancies; wave 2-3: 379 of 595 pregnancies; wave 3-4: 162 of 260 pregnancies) were among those with a low DAP score, approximately one-quarter (weighted percentages, 24%-28%; wave 1-2: 146 of 633 pregnancies; wave 2-3: 153 of 595 pregnancies; wave 3-4: 64 of 260 pregnancies) were among those with a midrange DAP score, and a weighted 13% to 21% (wave 1-2: 105 of 633 pregnancies; wave 2-3: 63 of 595 pregnancies; wave 3-4: 34 of 260 pregnancies) were among those with high DAP scores. Over each annual wave, a weighted 4% to 6% of included participants experienced a low DAP pregnancy (wave 1-2: 382 of 7396 participants; wave 2-3: 379 of 7488 participants; wave 3-4: 162 of 3719 participants), 2% to 3% experienced a midrange DAP pregnancy (wave 1-2: 146 of 7396 participants; wave 2-3: 153 of 7488 participants; wave 3-4: 64 of 3719 participants), and 1% to 2% experienced a high DAP pregnancy (wave 1-2: 105 of 7396 participants; wave 2-3: 63 of 7488 participants; wave 3-4: 34 of 3719 participants).

## Discussion

Using state-representative, longitudinal data collected among reproductive-aged females in 9 diverse US states, this cohort study found that pregnancy preferences were associated with incident pregnancy over 1 year; those with the greatest desire to avoid pregnancy had the lowest estimated probability of incident pregnancy. Significant differences in the association of desire to avoid pregnancy with incident pregnancy were found by sociodemographic characteristics, indicating the degree to which individuals attain their pregnancy preferences differs based on age, parity, prior-year birth, cohabitation with romantic partner, education level, employment, and racial and ethnic identity.

Interpreting the meaning of pregnancies occurring or not occurring across levels of DAP scores is inherently less straightforward as for predefined pregnancy desire and timing categories, like those in the NSFG. Central to the development of the DAP scale is the notion that some people do not hold clear desires about pregnancy and childbearing, and the idea of intending or planning a pregnancy does not resonate universally, particularly among those who are uncertain about their futures or for whom having a child under normative circumstances is inaccessible. While women with low DAP scores respond to DAP items with a clearly positive orientation to pregnancy (eg, agreement that a baby would be a positive addition to their lives, disagreement it would be bad for their lives), they are not necessarily actively trying to become pregnant, making it less straightforward to ascribe meaning to lack of pregnancy in this group.

In our data, approximately one-quarter of pregnancies were among participants with midrange DAP scores. Although qualitative research has documented the range of preferences people have about a potential pregnancy, including uncertainty, ambivalence, fatalism, and indifference,^22,23^ explicit categories for these orientations are only beginning to be offered as response options, including in the NSFG.^1^ In 2015, for approximately 2% of births and 7% of abortions, the respondent retrospectively reported their feelings about the pregnancy in a way that did not fit a clear preference to have or avoid a pregnancy.^1^ Our results suggest these proportions may be underestimates, stemming in part from the fact that survey respondents likely fit themselves into a clear pregnancy desire category when offered. Furthermore, people’s retrospective pregnancy desire assessments likely incorporate feelings they had after pregnancy discovery, when the notion of childbearing is more concrete, and feelings are likely more manifest and certain.

A substantial advantage of prospective over retrospective pregnancy desire data is the availability of preference data among nonpregnant people. Using retrospective designs, pregnancy desire or preference data are unavailable for those who did not experience pregnancy, so researchers are limited to calculating, for instance, undesired pregnancy rates among all reproductive-aged women (rather than among women who did not desire pregnancy).^1,21^ Our prospective data allow us to differentiate differences in the distribution of pregnancy preferences from differences in incident pregnancy by preference level and thus more accurately identify factors associated with not attaining one’s pregnancy preference.^3^ For age, parity, and prior-year birth, we found consistent differences in the probability of pregnancy across all DAP groups. Participants aged 18 to 34 years were most likely to experience pregnancy across all levels of pregnancy preferences; pregnancy declined with increasing age across all DAP groups. Similarly, pregnancy was generally elevated across all DAP levels for primiparous (vs nulliparous or multiparous) participants and those who had a recent birth. These results align with fecundability research showing that younger individuals and those who have previously given birth are more likely to experience a pregnancy.^24,25^ Social and family circumstances, as well as 2-child norms and birth spacing preferences, may contribute to higher probability of pregnancy among primiparous individuals. Behavioral factors, like lower contraceptive use postpartum, may explain increased probability of pregnancy among those with a prior-year birth.^26^

The patterns of pregnancy we found by race and ethnicity and education level are suggestive of inequities in attainment of reproductive preferences. Among those with the greatest openness to pregnancy, White, non-Hispanic participants were more likely to experience pregnancy compared with Black, non-Hispanic and Hispanic participants. The inverse was true among those with the greatest desire to avoid pregnancy. Among those with high DAP scores, participants with more education were less likely to experience pregnancy. Research has identified factors that may impede attainment of pregnancy preferences for racially and ethnically minoritized and low-resource individuals, including structural barriers to contraceptive use,^27^ preferred method use,^28^ abortion,^29^ and fertility treatment,^30^ as well as attitudinal considerations, such as cultural or religious beliefs^31^ and mistrust of the health care system.^32^ Attention to helping racially and ethnically minoritized individuals in the US both avoid pregnancy and achieve pregnancy when preferred are needed to address reproductive inequities.

Although not directly comparable, our findings are consistent with population-based estimates that use birth certificate data, facility reporting of abortions, and the NSFG retrospective categorizations of pregnancy intention. In 2015, an estimated 3.6 pregnancies per 100 reproductive-aged women overall occurred too soon or were not wanted, and an estimated 5.2 pregnancies per 100 reproductive aged women occurred at about the right time or later than wanted.^1^ These figures align roughly with our approximately 1 to 2 high DAP pregnancies, 2 to 3 midrange DAP pregnancies, and 4 to 6 low DAP pregnancies per 100 participants per year. Notably, denominators for our estimates exclude participants who were already pregnant, infertile, or had a tubal ligation, while NSFG denominators included all reproductive-aged women.

### Limitations

This study has limitations. The Surveys of Women relied on self-report of incident pregnancy, and it is likely that pregnancies were underreported, particularly those ending in abortion.^33^ We likely underestimated pregnancy proportions among those with high DAP scores, and results could be inaccurate to the extent that underreporting was differential by participant characteristics. A small number of participants experienced more than 1 pregnancy in 1 year, so our annual probabilities of pregnancy are slightly underestimated. The DAP scale items refer to preferences about a potential pregnancy within 3 months and childbearing within 1 year. Preferences change over time, so misclassification of the DAP scores by pregnancy status could have occurred.

Because the Surveys of Women were administered over different years across 9 states, results cannot be generalized to any particular year nor account for any fluctuations in pregnancy preferences or incident pregnancy between 2017 and 2023. Although we employed weights for attrition, results do not account for differential attrition by unmeasured characteristics not employed to generate the weights. Although geographically diverse, the 9 study states were not randomly selected, so results cannot be generalized to all reproductive-aged females in the US.

## Conclusions

To our knowledge, this is the first population-based, longitudinal cohort study to employ a rigorously developed measure of pregnancy and childbearing preferences. By capturing preferences across different conceptual domains and not requiring participants to specify a clear pregnancy desire, this measurement approach is likely to more accurately and precisely capture individuals’ true feelings about pregnancy than standard approaches to pregnancy orientation measurement. Our use of prospective data allows us to calculate incident pregnancy by pregnancy preference grouping, an approach that can better identify factors associated with not attaining one’s preference. Our findings identify areas of inequity in reproductive autonomy because individuals’ attainment of their pregnancy preferences differed by sociodemographic characteristics like racial and ethnic identity and education level.

## Notes

Supplement 1. eTable 1. Surveys of Women Survey Administration Timing by State and Wave eTable 2. Unadjusted Predicted Probabilities of Incident Pregnancy (and 95% Confidence Intervals) Over a Year, by Desire to Avoid Pregnancy (DAP) Scale Score and State, and P-Values for Differences by State Within DAP Score Grouping eAppendix. Supplementary Interpretation of Table 2.

Supplement 2. Data Sharing Statement
