# Supplementary material for: Pregnancy Preferences and Incident Pregnancy in the US
Source: JAMA Netw Open. 2025 Oct 9;8(10):e2536697. doi: 10.1001/jamanetworkopen.2025.36697 (PMC12511997; doi:10.1001/jamanetworkopen.2025.36697)
Supplement: Supplement 1. — eTable 1. Surveys of Women Survey Administration Timing by State and Wave eTable 2. Unadjusted Predicted Probabilities of Incident Pregnancy (and 95% Confidence Intervals) Over a Year, by Desire to Avoid Pregnancy (DAP) Scale Score and State, and P-Values for Differences by State Within DAP Score Grouping eAppendix. Supplementary Interpretation of Table 2. [file jamanetwopen-e2536697-s001.pdf]

Supplemental Online Content

Bullington BW, Muñoz I, Boscardin WJ, Rocca CH. Pregnancy preferences and incident pregnancy in the US. JAMA Netw. Open. 2025;8(10):e2536697. doi:10.1001/jamanetworkopen.2025.36697

eTable 1. Surveys of Women Survey Administration Timing by State and Wave

eTable 2. Unadjusted Predicted Probabilities of Incident Pregnancy (and 95% Confidence Intervals) Over a Year, by Desire to Avoid Pregnancy (DAP) Scale Score and State, and P-Values for Differences by State Within DAP Score Grouping

eAppendix. Supplementary Interpretation of Table 2

© 2025 Bulington BW et al. *JAMA Network Open*.

**eTable 1.** Surveys of Women survey administration timing by state and wave								
**State**	**Wave**	**Year fielded**						
		2016	2017	2018	2019	2020	2021	2022
**Alabama**	1							
	2							
	3							
	4							
**Arizona**	1							
	2							
	3							
**Delaware**	1	*	*					
	2							
	3							
**Iowa**	1							
	2							
	3							
	4							
**Maryland**	1	*	*					
	2							
	3							
**New Jersey**	1							
	2							
	3							
**Ohio**	1							
	2							
	3							
	4							
**South Carolina**	1							
	2							
	3							
	4							
**Wisconsin**	1							
	2							
	3							

*The DAP Scale was not included in Delaware and Maryland wave 1 surveys, so these surveys were not included in analyses.

© 2025 Bulington BW et al. *JAMA Network Open*.

eTable 2. Unadjusted model probabilities of incident pregnancy (and 95% confidence intervals) over a year,[1] by Desire to Avoid Pregnancy (DAP) Scale score and state, and p-values for differences by state within DAP score grouping

	**Low DAP score[2]**		**Mid-range DAP score[3]**		**High DAP score[4]**	
	**Probability (95% CI)**	**p**	**Probability (95% CI)**	**p**	**Probability (95% CI)**	**p**
**State**						
Alabama	0.16 (0.12, 0.21)	ref	0.06 (0.04, 0.09)	ref	0.03 (0.01, 0.04)	ref
Arizona	0.21 (0.15, 0.27)	p=0.23 vs. AL	0.09 (0.05, 0.13)	p=0.34 vs. AL	0.03 (0.02, 0.05)	p=0.63 vs. AL
Delaware	0.21 (0.11, 0.31)	p=0.42 vs. AL<br>p=0.98 vs. AZ	0.03 (0.00, 0.07)	p=0.23 vs. AL<br>p=0.10 vs. AZ	0.03 (0.00, 0.05)	p=0.95 vs. AL<br>p=0.68 vs. AZ
Iowa	0.30 (0.24, 0.36)	**p=0.001 vs. AL**<br>**p=0.04 vs. AZ**<br>p=0.16 vs. DE	0.07 (0.04, 0.09)	p=0.99 vs. AL<br>p=0.36 vs. AZ<br>p=0.24 vs. DE	0.03 (0.01, 0.04)	p=0.80 vs. AL<br>p=0.42 vs. AZ<br>p=0.91 vs. DE
Maryland	0.31 (0.17, 0.44)	**p=0.03 vs. AL**<br>p=18 vs. AZ<br>p=0.26 vs. DE<br>p=0.96 vs. IA	0.06 (0.01, 0.12)	p=0.98 vs. AL<br>p=0.52 vs. AZ<br>p=0.33 vs. DE<br>p=0.98 vs. IA	0.01 (0.00, 0.03)	p=0.33 vs. AL<br>p=0.21 vs. AZ<br>p=0.41 vs. DE<br>p=0.39 vs. IA
New Jersey	0.29 (0.22, 0.36)	**p=0.003 vs. AL**<br>p=0.08 vs. AZ<br>p=0.21 vs. DE<br>p=0.88 vs. IA<br>p=0.89 vs. MD	0.09 (0.05, 0.14)	p=0.26 vs. AL<br>p=0.87 vs. AZ<br>p=0.08 vs. DE<br>p=0.28 vs. IA<br>p=0.44 vs. MD	0.04 (0.02, 0.06)	p=0.22 vs. AL<br>p=0.43 vs. AZ<br>p=0.35 vs. DE<br>p=0.11 vs. IA<br>p=0.10 vs. MD
Ohio	0.24 (0.19, 0.29)	**p=0.04 vs. AL**<br>p=0.45 vs. AZ<br>p=0.60 vs. DE<br>p=0.13 vs. IA<br>p=0.36 vs. MD<br>p=0.22 vs. NJ	0.08 (0.05, 0.10)	p=0.50 vs. AL<br>p=0.67 vs. AZ<br>p=0.13 vs. DE<br>p=0.53 vs. IA<br>p=0.68 vs. MD<br>p=0.54 vs. NJ	0.03 (0.02, 0.03)	p=0.69 vs. AL<br>p=0.33 vs. AZ<br>p=0.84 vs. DE<br>p=0.89 vs. IA<br>p=0.42 vs. MD<br>p=0.07 vs. NJ

© 2025 Bulington BW et al. *JAMA Network Open*.

South Carolina	0.24 (0.17, 0.30)	**p=0.04 vs. AL**<br>p=0.43 vs. AZ<br>p=0.57 vs. DE<br>p=0.17 vs. IA<br>p=0.39 vs. MD<br>p=0.27 vs. NJ<br>p=0.94 vs. OH	0.10 (0.06, 0.14)	p=0.10 vs. AL<br>p=0.62 vs. AZ<br>p=0.04 vs. DE<br>p=0.12 vs. IA<br>p=0.30 vs. MD<br>p=0.76 vs. NJ<br>p=0.28 vs. OH	0.04 (0.02, 0.07)	p=0.32 vs. AL<br>p=0.55 vs. AZ<br>p=0.41 vs. DE<br>p=0.19 vs. IA<br>p=0.91 vs. MD<br>p=0.12 vs. NJ<br>p=0.13 vs. OH
Wisconsin	0.30 (0.24, 0.36)	**p=0.001 vs. AL**<br>p=0.05 vs. AZ<br>p=0.18 vs. DE<br>p=0.92 vs. IA<br>p=0.92 vs. MD<br>p=0.95 vs. NJ<br>p=0.15 vs. OH<br>p=0.21 vs. SC	0.07 (0.04, 0.11)	p=0.68 vs. AL<br>p=0.63 vs. AZ<br>p=0.17 vs. DE<br>p=0.69 vs. IA<br>p=0.77 vs. MD<br>p=0.52 vs. NJ<br>p=0.89 vs. OH<br>p=0.31 vs. SC	0.02 (0.01, 0.04)	p=0.72 vs. AL<br>p=0.44 vs. AZ<br>p=0.82 vs. DE<br>p=0.86 vs. IA<br>p=0.50 vs. MD<br>p=0.17 vs. NJ<br>p=0.93 vs. OH<br>p=0.23 vs. SC

[1] In N=167 (0.9% of observations) multiple pregnancies over the course of one year were reported. Pregnancies ending in abortion may have been underreported.
[2] Low DAP score: 0 to 1.5.
[3] Mid-range DAP score: >1.5 to 2.5.
[4] High DAP score: >2.5 to 4.

© 2025 Bulington BW et al. *JAMA Network Open*.

eAppendix. Supplementary Interpretation of Table 2

Age

When comparing 35 to 39 years with 25 to 29 years, there were significant differences for low (*P* = .001), mid-range (*P* < .001), and high (*P* = .001) DAP scores.

When comparing 35 to 39 years with 30 to 34 years, there were significant differences for low (*P* = .006), mid-range (*P* < .001), and high (*P* < .001) DAP scores.

When comparing 40 to 47 years with 25 to 29 years, there were significant differences for low (*P* < .001), mid-range (*P* < .001), and high (*P* < .001) DAP scores.

When comparing 40 to 47 years with 30 to 34 years, there were significant differences for low (*P* < .001), mid-range (*P* < .001), and high (*P* < .001) DAP scores.

When comparing 40 to 47 years with 35 to 39 years, there were significant differences for low (*P* = .01), mid-range (*P* = .002), and high (*P* = .05) DAP scores.

Parity

When comparing a parity of 2 with a parity of 1, there was a significant difference for high DAP scores (*P* = .001).

When comparing a parity of 3 with a parity of 1, there were significant differences for low (*P* < .001) and high (*P* = .03) DAP scores.

When comparing a parity of 3 with a parity of 2, there was a significant difference for low DAP scores (P = .004).

When comparing a parity of 4 with a parity of 2, there was a significant difference for high DAP scores (*P* = .04).

Highest education

When comparing Bachelor’s degree or more with some college, there were significant differences for low (*P* = .002) and high (*P* = .004) DAP scores.

© 2025 Bulington BW et al. *JAMA Network Open*.

Employment

When comparing out of work force with unemployed, there was a significant difference for high DAP scores (*P* = .001).

Race and ethnicity

When comparing Asian, non-Hispanic with Black non-Hispanic (*P* = .001), Hispanic (*P* = .02), and multiracial or another race (P=.003) there were significant differences for mid-range DAP scores.

When comparing multiracial or another race with Black, non-Hispanic (*P* = .005) and Hispanic (*P* = .03), there were significant differences for high DAP scores.

© 2025 Bulington BW et al. *JAMA Network Open*.
