# Supplementary material for: Pregnancy Preferences and Incident Pregnancy in the US
Source: JAMA Netw Open. 2025 Oct 9;8(10):e2536697. doi: 10.1001/jamanetworkopen.2025.36697 (PMC12511997; doi:10.1001/jamanetworkopen.2025.36697)
Supplement: Supplement 2. — Data Sharing Statement [file jamanetwopen-e2536697-s002.pdf]

Data Sharing Statement

Bullington. Pregnancy Preferences and Incident Pregnancy in the US. *JAMA Netw Open*. Published October 09, 2025. doi:10.1001/jamanetworkopen.2025.36697

Data
Data available: No

Additional Information
Explanation for why data not available: The Surveys of Women data were made available to the research team through a Data Use Agreement with NORC, the custodian of the data. As such the research team is not at liberty to distribute data.
